# Molecular characterization of hepatitis B virus among chronic hepatitis B patients from Pointe Noire, Republic of Congo

**DOI:** 10.1186/s13027-016-0088-3

**Published:** 2016-09-15

**Authors:** Brunel Monic Angounda, Gildas Hoffman Ngouloubi, Amélia Bokilo Dzia, Luc Magloire Anicet Boumba, Warda Baha, Donatien Moukassa, Gabriel Ahombo, Moulay Mustapha Ennaji, Jean-Rosaire Ibara

**Affiliations:** 1 Laboratory of screening of the transmitted infectious diseases, National Blood Transfusion Centre, Brazzaville, Republic of Congo; 2 Cellular and Molecular Biology Laboratory, Faculty of Science and Technology, University Marien NGOUABI, Brazzaville, Congo; 3 Laboratory of Virology, Microbiology, Quality and Biotechnologies/Eco-toxicology and biodiversity, Virology Team, Cancerology, Quality and Medical Biotechnology, Faculty of Science and Techniques Mohammedia University Hassan II of Casablanca, BP: 146, Mohammedia, 20650 Morocco; 4 Gastroenterology, Adolphe Sice Hospital, Pointe Noire, Republic of Congo; 5 Faculty of Health Sciences, University Marien NGOUABI, Brazzaville, Congo; 6 General Laondjili Hospital, Pointe-Noire, Congo

**Keywords:** Hepatitis B Virus, Chronic, Genotypes, Phylogenetics

## Abstract

**Background:**

Chronic Hepatitis B infection is a major health problem in Republic of Congo therefore molecular analysis of HBV strains is important to detect the patients at high risk of disease progression.

**Methods:**

Serum samples were obtained from 111 chronic HBV patients in Pointe Noire. HBsAg, HBeAg and HBeAb were detected. A fragment of the preS1 region of HBV was amplified and sequenced to determine genotypes, subgenotypes and to identify mutations.

**Results:**

Of the 111 samples analyzed, 35 patients were asymptomatic carriers (ASC), 24 with a chronic active hepatitis (CAH), 33 with liver cirrhosis (LC) and 19 have a hepatocellular carcinoma (HCC). The mean age were 45 ± 13 year, 88 (79.3 %) were male and 23 (20.7 %) female. The prevalence of HBeAg was 15.3 % and 73 % of subjects were anti-HBe positive. The mean serum level of alanine aminotransferase transaminase (ALT) and aspartate transaminase (AST) was 25.1 ± 9 IU/L and 28.6 ± 10 IU/L respectively. Eighty two samples out of 111 (73.9 %) were genotyped by the analyzing of the S region of HBV, 58 (70.7 %) cases belonged to HBV genotype E and 24 (29.3 %) were genotype A with three subgenotypes; A3 (66.7 %), A4 (20.8 %) and A6 (12.5 %). Prevalence of genotype A was relatively high in CAH (33.3 %) and HCC (31.6 %) patients in comparison with other groups. The most prevalent amino acids substitutions were R38K found in 14 (17.1 %) sequences, following by H44L in 11 (13.4 %), K13E in 8 (9.8 %), N29K in 8 (9.8 %), A35E in 8 (9.8 %), V80I in 7 (8.5 %) and in 6 (7.3 %) sequences for S90T. Different substitutions located in the hepatocyte binding site were higher among patients with LC and HCC (*p* < 0.05).

**Conclusions:**

This study have shown that HBV genotype E and A were the most frequent strains circulating in Republic of Congo patients. HBV pres1 substitutions found in this study were associated with severe clinical forms of liver diseases. This data have shown the importance of implementing an effective program to fight HBV infection.

## Background

Hepatitis B virus (HBV) infection varies from an acute and chronic phase liver diseases with progression to cirrhosis and hepatocellular carcinoma (HCC) [1]. HBV is a serious public health problem, nearly two billion people worldwide were infected and more than 350 million people live with chronic infection [2]. HBV is a partially double-stranded circular DNA virus and has high genetic variability. Currently, there are ten genotypes designated A to J, based on an intra-group nucleotide divergence of up to 4.2 % of the S-genome sequences or in >8 % of the entire genome sequences [3]. These genotypes have a specific geographic distribution worldwide [4]. HBV genotypes A and D have global distributions, genotypes B and C are endemic in Asia; genotype E originates from West and sub-Saharan Africa, genotypes F and H are restricted in Central and South America, genotype G has been reported in Europe and North America; two new genotypes I and J, identified respectively in South-East Asia and Japan [5–8]. In Africa area, the management of chronic hepatitis B is a major health problem, due to the late detection of infection and lack of effective monitoring means [5]. HBV contains a polymerase enzyme without proofreading activity, high error frequencies on RNA or DNA copying are selected during replication phase, resulting in mutations that play a substantial role in determination of the clinical pathogenesis degree [9, 10]. Recently, several studies have reported the presence of mutations in pre-S, basal core promoter (BCP) and the precore (PC) regions are highly correlated with the severity of HBV-related liver diseases, including fibrosis, cirrhosis and HCC [11–13]. Moreover, several mechanisms are reported to be associated with fulminate hepatitis B which includes specifically mutations in the major hydrophilic loop or in the “a” determinant region of S gene [14, 15]. Many studies reported that Pre-S mutants lead accumulation of large envelope proteins in the endoplasmic reticulum (ER), resulting in ER stress associated with aggravated liver disease [16, 17]. Republic of Congo is classified as having high endemicity of HBV [18] with a prevalence of Hepatitis B surface antigen (HBsAg) ranges from 6.5 % to 11.4 % [19–22]. The average length of survival is low due to stage of gravity and to late hospitalization of patients [23]. This virus is considered as the most common cause of cirrhosis (63 %) and HCC (73.5 %) in Pointe Noire and Brazzaville [24, 25]. Despite all this data, none molecular study have been performed in Republic of Congo. Thus, this first work aimed to characterize the different strains of HBV in chronic carriers; it might constitute an effective basis to fight against this infection.

## Methods

### Patients and serum samples

This is a cohort study, conducted in chronic HBV patients followed in the pathology department at the hospital Adolphe Sice in Pointe Noire, during the period from February to September 2014. Serum samples were obtained from 111 patients with chronic HBV infection and all patients were known to have been positive for HBsAg during the previous 6 months. The demographic data including gender, age and biochemical data such as alanine aminotransferase transaminase (ALT) and aspartate transaminase (AST) levels were also available for these patients. Four groups of patients were classified according to their clinical diagnosis based on liver biochemical tests, hepatitis virus markers, alfa-fetoprotein (AFP) and ultrasonography, it consists in: 35 patients with asymptomatic carriers (ASC), 24 patients with chronic active hepatitis (CAH), 33 with liver cirrhosis (LC) and 19 patients with HCC. Five milliliter of blood was collected from each participant and the serum was separated and stored at -70 °C until used.

Informed consent was obtained from all participating subjects. The study protocol conformed to the Declaration of Helsinki, and was approved by the Ethics Committee of Research in Health Sciences in Republic of Congo.

### Serologic test for HBV markers

All serum samples were tested for HBsAg (Monolisa™ HBsAg ULTRA), hepatitis B e antigen (HBeAg) and hepatitis B e antibody (Monolisa™ HBe Ag-Ab PLUS) using an enzyme linked immunosorbent assay (ELISA) according to the manufacturer’s instructions (Monolisa, Bio-Rad Laboratories, Marnes La Coquette).

### DNA Extraction

We used a standard method of phenol/chloroform to extract DNA [26], 400 μl of serum was mixed with 300 μl of lysis buffer (1 M Tris-HCl, pH 8.0, 0.2 M EDTA, 5 % SDS and 5 M NaCl) in the presence of 5 μl of 20 mg/ml proteinase K (Promega Corp., WI, USA) and incubated at 56 °C for 2 h. Then DNA was precipitated with 2/5 volumes of 7.4 M ammonium acetate and 2 volumes of 96 % ethanol. The DNA was resuspended in RNase free water and stored at –20 °C until use.

### HBV DNA amplification and sequencing of the preS1 region

Detection of HBV DNA was performed by a nested PCR and amplifying of the fragments covering the preS1 gene of HBV with two consensus primers. The sequence of the PCR primers were HBPr1 (nt 2850–2868,5’-GGGTCACCATATTCTTGGG-3’) and HBPr135 (nt803-822, 5’- CAAGACAAAAGAAAATTGG-3’) for the outer primer pair and HBPr2 (nt 2867–2888, 5’-GAACAAGAGCTACAGCATGGG-3’) and HBPr3 (nt 1547–1569, 5’- CCACTGCATGGCCTGAGGATG-3’) for the inner one, as previously described by Stuyver et al. [7]. Briefly, the first round PCR was performed in a 20 μl mixture containing 5 μl DNA, 10 mM of dNTPs, 2.5 mM of MgCl2, 1X colorless GoTaq® Flexi Buffer and 10 mM of each primer, 0.2 U of GoTaq® DNA Polymerase (Promega, Madinson, USA). For the second round PCR, 2 μl of the first round PCR product was used as DNA template. For the both the first and second round, the thermocycling profile consisted of AmpliTaq activation at 94 °C for 5 min, followed by 35 cycles of PCR amplification using the following temperatures: denaturation at 94 °C for 30 s, annealing at 50 °C for 30 s and extension at 72 °C for 30 s, with a final elongation at 72 °C for 7 min. All PCR amplification were performed in a Perkin Elmer 2400 GeneAmpR® PCR thermal Cycler (Scientific Support, Inc, Hayward, CA). The products were analyzed on a 2 % agarose gel and visualized by staining with ethidium bromide.

### Phylogenetic analysis

HBV genotypes were determined using phylogenetic analysis. All HBV sequences were aligned with corresponding sequences of HBV belonging to genotype A-J obtained from GenBank database (http://www.ncbi.nih.gov). Multiple sequence alignment was edited by Bioedit software [27] and subsequently manually edited to remove gaps or ambiguously aligned sites. The phylogenetic tree was constructed using the neighbor-joining method by MEGA software version 6.0.5 [28] and the reliability of the tree was evaluated by means of bootstrap analysis with 1000 replicates. Pairwise evolutionary distances were computed using the Kimura two parameter model.

### Nucleotide sequence accession numbers

The nucleotide sequence data reported in this paper have been submitted to the GenBank databases under accession numbers KX130002 - KX130083.

### Statistical analysis

Statistical tests were performed using EpiInfo software version 7.0 (Centers for Disease Control and Prevention and World Health Organization, Geneva, Switzerland). Data were expressed as mean ± SD and percentages. Comparisons between groups were analyzed by chi-square test and p-value of less than 0.05 was considered statistically significant.

## Results

In overall, 111 chronic patients were enrolled in this study. The mean age of patients was 45 ± 13 (range, 21 to 68) year, of that, 88 (79.3 %) patients were male and 23 (20.7 %) female. The prevalence of HBeAg was 15.3 % and 73 % of subjects were anti-HBe positive. The presence of HBeAg was high in CAH (41.18 %) and HBeAb positive was higher in the age group 39-45 year with a prevalence of 56.7 %. The mean serum level of ALT and AST was 25.1 ± 9 IU/L and 28.6 ± 10 IU/L respectively. The ALT and AST was significantly different among CAH and other clinical status (Table 1). However, no correlation was found in the transaminases levels regarding the HBe status and clinical diagnosis (*P* > 0.05).

**Table 1:** Distribution of demographic and clinical characteristics of chronic hepatitis B patients in Pointe Noire, Republic of Congo (2014)

Characteristics	Clinical Status			
	ASC (35)	CAH (24)	LC (33)	HCC (19)
Gender				
Male n(%)	28 (80)	19(79.2)	27 (81.8)	14(73.7)
Female n(%)	7 (30)	5 (20.8)	6(18.2)	5 (26.3)
Age (%)				
21-39	11 (31.4)	13 (54.2)^a^	8 (24.2)	3 (15.8)
40-55	20 (57.1)^a^	5 (20.8)	15 (45.5)	7 (36.8)
56-68	4 (11.4)	6 (25)	10 (30.3)	9 (47.4)
ALT (IU/l), mean ± SD	15.8 ± 3	34.4 ± 6	25.6 ± 7	29.4 ± 5
AST (IU/l), mean ± SD	20.8 ± 7	42.1 ± 8	28.3 ± 6	26.5 ± 4
HBeAg positive(%)	2 (5.7)	7 (29.2)^a^	5 (15.2)	3 (15.8)
HBeAb positive(%)	28 (80)	14 (58.3)	24 (72.7)	15 (78.9)
HBV genotype(%)				
HBV/A	6 (17.1)	8 (33.3)	4 (12.1)	6 (31.6)
HBV/E	18 ((51.4)	11 (45.8)	21 (63.6)	8 (42.1)

*ASC* asymptomatic carriers, *CAH* chronic active hepatitis, *LC* liver cirrhosis, *HCC* hepatocellular carcinoma (HCC), *a* significant difference

**Fig. 1:**
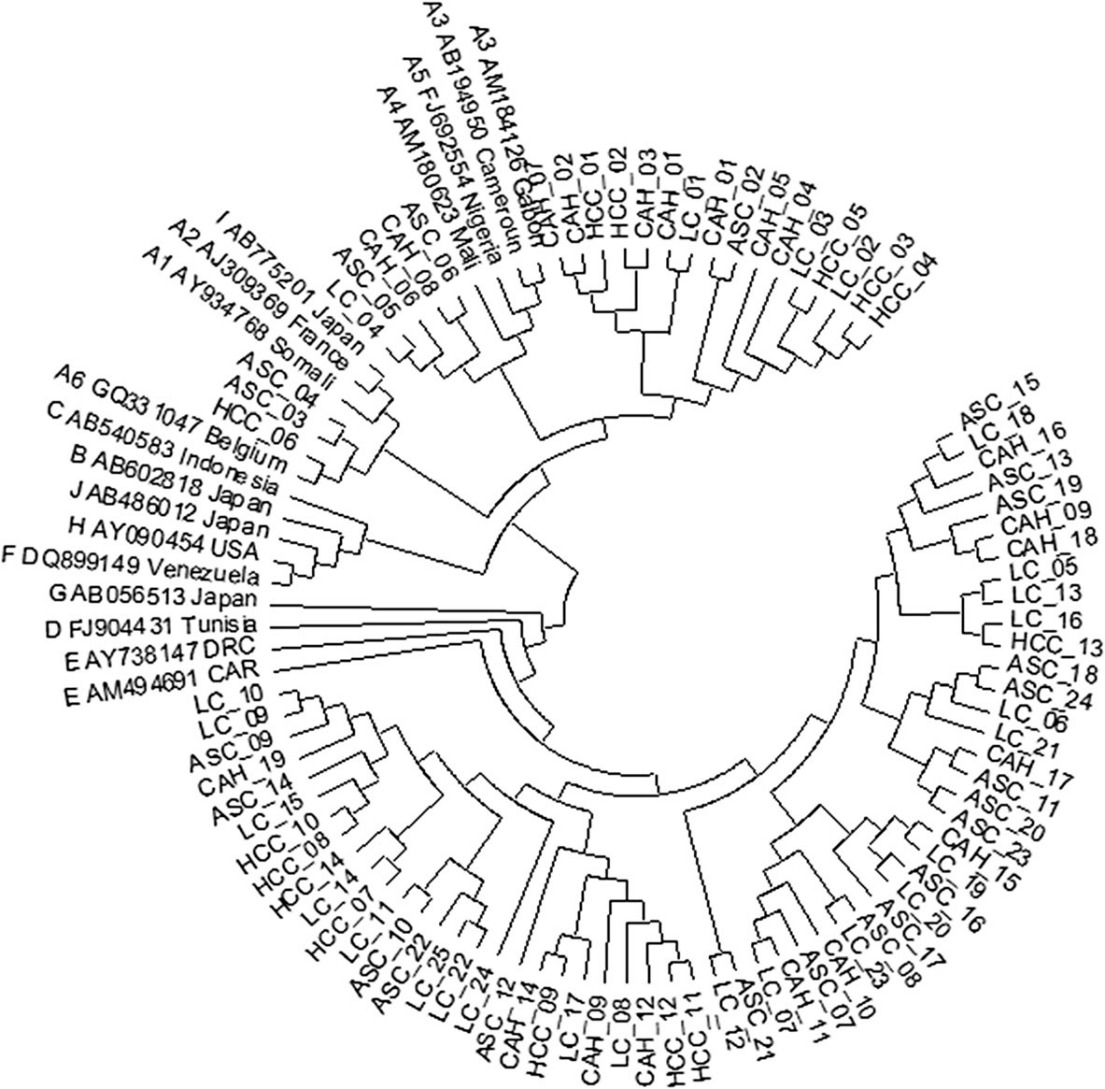
Phylogenetic analysis was based on nucleotide sequencing on the preS1 region of HBV. The sequences of the Congolese patients were compared with representative sequences of all HBV genotypes and subgenotypes (A to I) retrieved from GenBank database. The sequences are labeled by their accession numbers and country, [CAR, Central African Republic; DRC, Democratic Republic of Congo]. The phylogenetic tree was generated by MEGA 6.0 program using the neighbor-joining method and bootstrap values were obtained from 1000 replicates

Eighty two samples out of 111 (73.9 %) were genotyped in the HBV S region. HBV DNA was detected in 68.6 % (24/35) of ASC, 79.2 % (19/24) of CAH, 76.8 % (25/33) of LC and 73.7 % (14/19) of HCC. Of these, 58 (70.7 %) subjects were infected with HBV genotype E and 24 (29.3 %) were infected with HBV genotype A. The subgenotypes of these 24 HBV/A isolates were further determined by comparison with references sequences, 16 strains belonged to subgenotypes A3 (66.7 %), 5 to A4 (20.8 %) and 3 were subtype A6 (12.5 %) (Fig. 1). The Association between liver disease and the prevalence of HBV genotypes have shown that, an overall predominance of HBV genotype E. HBV genotype A was higher in CAH (33.3 %) and HCC (31.6 %) patients. There was no significant difference between age and biochemical parameters of liver disease according to different genotypes (*p* > 0.05).

### Frequency and distribution of mutations in the preS1 region

**Fig. 2:**
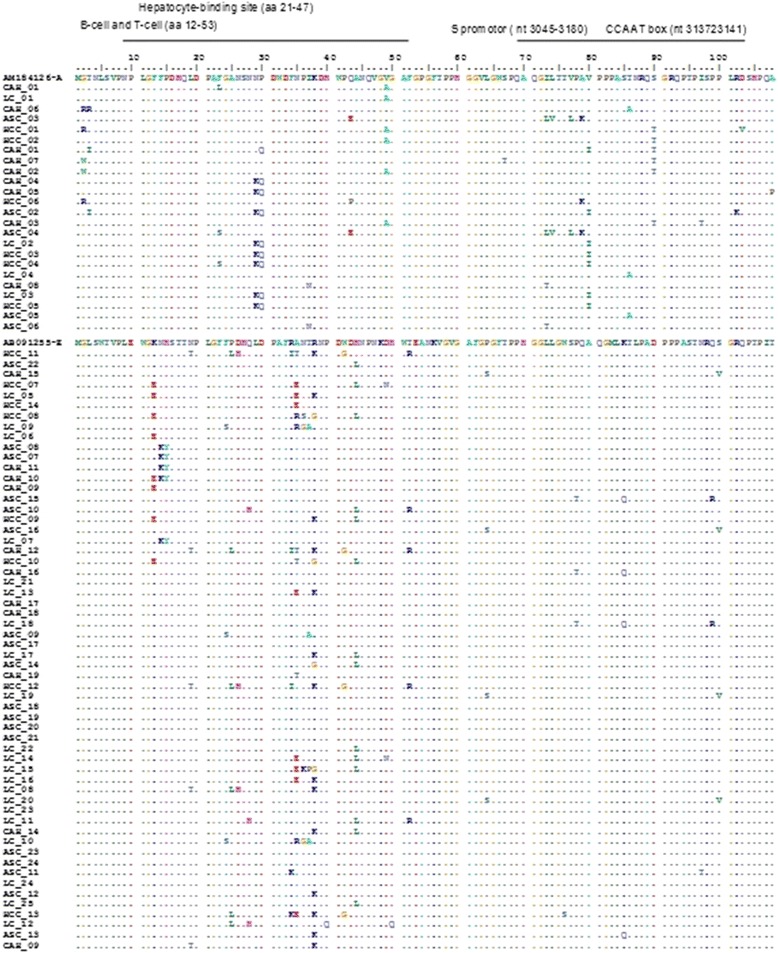
Alignments of deduced amino acid sequences of pre-S1 from OBI strains compared to the consensus sequence of genotype E (AB091255) and genotype A (AM184126) retrieved from GenBank database. The amino acids were identified by single letter code. Dots represent identical amino acids with the reference consensus sequences for genotype E or A shown on top of each sequence. The immune epitopes corresponding to B-and T-cell (aa12-53), hepatocyte binding site (aa 21-47), S promoter (nt 3045-3180) and CCAAT box (nt 3137-3141) are marked correspondingly at the top of the alignment. aa, amino acid; nt, nucleotide

Among the 82 (73.9 %) strains sequenced in pres1 region, different amino acid substitutions were detected (Fig. 2). The most prevalent variant were R38K found in 14 (17.1 %) sequences, following by H44L in 11 (13.4 %), K13E in 8 (9.8 %), N29K in 8 (9.8 %), A35E in 8 (9.8 %), V80I in 7 (8.5 %) and in 6 (7.3 %) sequences for S90T (Table 2).

**Table 2:** Distribution of the PreS1 mutation prevalent among chronic hepatitis B patients according to clinical status

Amino acid substitutions	Number of isolates(%)	Genotype	ASC	CAH	LC	HCC	Type of Epitope
K13E	8	E	0	2	2	4	
N14K	5	E	1	2	2	0	
H15Y	5	E	1	2	2	0	
N19T	5	E	0	2	1	2	B and T-cell
P25L	6	E	0	1	1	2	&
N29K	8	A	1	2	2	3	Hepatocyte-binding site
P30Q	7	A	2	2	2	3	
A35E	8	E	0	0	5	3	
R38K	14	E	2	3	5	4	
R38G	4	E	1	0	1	2	
W42G	4	E	0	1	0	3	
H44L	11	E	2	1	4	4	
V49A	6	A	0	3	1	2	
T52R	5	E	1	1	1	2	
P64S	4	E	1	1	2	0	S promoter (nt 3019-3201)
V80I	7	A	2	0	2	3	
K85Q	4	E	2	1	1	0	
S90T	6	A	1	3	0	2	
S100V	4	E	1	1	2	0	

*ASC* asymptomatic carriers, *CAH* chronic active hepatitis, *LC* liver cirrhosis, *HCC* hepatocellular carcinoma (HCC), *nt* nucleotide

The distribution of substitutions in pres1 region, shown 8.5 % (7/82) of mutations observed were located in B-cell and T-cell region and in hepatocyte binding site including R38K (17.1 %), H44L (13.4 %) and N29K (9.8 %). In addition, some other mutations were observed in the S promoter region and CCAAT box including V80I in 7 (8.5 %) cases and S90T in 6 (7.3 %) strains. These mutations were observed among different clinical groups; In the 11 sequences with R38K (17.1 %, 14/82) substitutions, high rate were observed in patients with LC (35.7 %, 5/14) and HCC (28.6 %, 4/14). The sequences with A35E and H44L amino acid substitutions were significantly more frequent in cases with LCC and HCC infection than in other patients groups (*P* < 0.05). There was no significant difference between substitutions V80I and S90T found in S promoter region with clinical status. Regarding preS1 mutations, these substitutions were low in ASC patients in comparison with LC and HCC subjects (*p* > 0.05). Furthermore, nearly 63 % of the HBV pre-S mutants were observed in patients over 40 years age, but no considerable difference was found in patients less than 39 years of age (*p* > 0.05) (Table 1).

## Discussion

Hepatitis B infection is hyperendemic in Republic of Congo and determination of genetic diversity has a vital interest in the characterization of the virus, liver damage, reaction to antiviral and evolution of infection.

In this present study, a relative young age (<30 years) of HBeAg carriers were observed. This finding is in agreement with other studies in Africa which indicate that perinatal transmission still persists with a significant risk of chronicity [29]. HBV DNA was found in 73.9 % of HBsAg patients, all these subjects were HBeAg positive and HBeAb was present in 58.6 % of samples. Indeed, some studies have reported that active HBV replication and a high ALT level may persist in patients with chronic HBV after HBeAg seroconversion [30]. Furthermore, chronic HBV patients with HBeAg negative have a risk of develop a severe form of liver disease that often progresses to cirrhosis and HCC [31].

The determination of HBV genotypes were based on amplification of the HBV S region, it has shown two main genotypes, HBV E (70.7 %) and A (29.3 %). This finding is in agreement with previous studies, which have reported that the genotype E is predominant in Africa and has rarely been found in other continents, except for a few cases mostly in individuals with an African background [6, 32–34]. HBV genotype A is found mainly in Northern and Western Europe, North America and Africa [5]. This genotype is subdivided into seven subgenotypes (A1 to A7) [35]. In this present study, we found the subgenotypes A3 (66.7 %), A4 (20.8 %) and A6 (12.5 %). The recent work carried out by Kurbanov et al., has detected the genotype A3 in Central and West Africa [36]. Some subgenotypes have been found in other countries; A4 was reported in Gambia and subgenotype A5 was reported in Nigeria and among African descendants in Haiti [37, 38]. Study conducted by Pourkarim et al., has found A6 strains from African-Belgian patients while A7 subtype was found by Hübschen et al in Rwanda and Cameroon [39, 40].

The low frequency of HBeAg positivity in both genotypes E and A found in this study, was reported in other studies in Africa [34, 41]. The study performed by Brichler et al., in Martinique, have reported that patients infected with HBV/A1 strains were more often HBeAg negative than those infected with genotype A2 or D [8]. In the other hand, Livingston et al., in Alaska have shown that the average age of HBeAg seroconversion to anti-HBe is lower in patients infected with genotypes A, B, D and F, as compared with genotype C [42]. Furthermore, it has reported that the anti-HBe seroconversion period is related to HBV genotypes; for genotype D, the median time was less than six years with a rapid evolution into HBe Ag negative mutant forms [29, 42].

In this study, HBV genotype E was predominant in all patient groups and HBV/A was relatively higher in CAH (33.3 %) and HCC (31.6 %) patients. Several studies performed in China, have shown a predominance of HBV genotypes C and B in patients with chronic liver disease, followed by genotypes D, E and A [43, 44]. The study carried in Indian subcontinent, demonstrated that the HBV genotypes D and F were associated with the genesis of HCC with a high morbidity [45]. Indeed, structural and functional differences between genotypes can influence the severity, complications and hepatitis B e antigen (HBeAg) seroconversion. HBV genotype E is described as being less linked to serious clinical manifestations than other genotypes with lower HBeAg positivity [34].

The phylogenetic analysis based on the sequencing of the HBV preS1 region have reported many substitutions; the most prevalent variant found were R38K in 14(17.1 %) sequences, following by H44L in 11(13.4 %) and K13E in 8(9.8 %) strains. Similar studies carried out in endemic countries, reported a HBV preS mutant prevalence have ranged from 0 % to 36 % in chronics HBV patients [9, 17, 46]. Our data show a significant high frequency of preS1 mutants in the LC and HCC subjects (*P* < 0.05). Indeed, several studies suggest that HBV preS mutations were associated with cirrhosis and the development of HCC [14, 47, 48].

In this work, 8.5 % of sequences have substitutions in the start codons, which can disrupt the synthesis of virions and impact HBV life cycle [49]. The major mutation found in epitope B-cell and T-cell and hepatocyte binding site were R38K (17.1 %), H44L (13.4 %) and N29K in 8 (9.8 %) sequences. This region plays an essential role in viral clearance and clinical recovery during natural infection [49]. Therefore, these mutations are determinant of viral persistence and may contribute further to the persistence of infection [50]. In the other hand, the main substitutions observed in the S promoter region and CCAAT box were V80I in 7(8.5 %) and S90T in 6(7.3 %) of sequences. A recent study performed by Yeung et al., has found deletion in S promoter region and CCAAT box in 38.1 % of cases and 16.7 %, respectively [14]. Another study performed by Sugauchi et al., detected the preS deletion mutants in 34 % patients with advanced liver disease and only 8 % of patients with clinical disease had a defect in the CCAAT box in the S promoter region [11].

Our study found no difference of mutation distribution according to HBe status; this is in agreement with the results from Pollicino et al., demonstrating that the emergence of preS/S mutants was comparable between HBeAg positive and HBeAg-negative patients [51]. We also found that preS mutations were more frequent in carriers of HBV genotype E than in those of genotype A. Indeed, mutations and evolution of HBV infection are different depending on genotypes and geographic location [11, 52]. A study performed in India, found a high prevalence of preS mutations among HBV carriers with genotype C (38.89 %) followed by HBV/A (33.33 %) and HBV/D (22.50 %) [17]. Furthermore, the studies carried in China among HBV carriers with progressive liver diseases, showed that preS deletion was more frequent in carriers of HBV genotype C than in those of genotype B [11, 50].

The high incidence of HBV preS1 mutations were found in LC (28.7 %) and HCC (41.6 %) patients group, followed by ASC (13.3 %) and ACH (16.4 %). In a previous study performed in China, the preS mutation rates were relatively low (2 %) in patients with acute HBV infection and a higher prevalence of preS1 deletions in HCC patients than in those with liver cirrhosis (43.1 % vs 35.2 %) [16]. In addition, Yeung et al. in Hong Kong have reported that patients with HCC had a higher prevalence of HBV with pre-S deletions than did patients without HCC (23 [33.3 %] of 69 vs 11 [15.9 %] of 69) [14]. The meta-analysis performed by Shijian et al. found that the PreS mutations were associated with 3.77-fold increased risk of HCC compared with HBV without mutations [53]. In fact, it has been reported that the preS mutants are involved in the mechanism of cytoplasmic retention of large surface protein. This accumulation can activate cellular grp78 and grp94 promoters by inducing endoplasmic reticulum stress and lead to oxidative DNA damage of HBV-infected hepatocytes [54]. Thus, the presence of oxidative DNA lesions stimulates DNA repair activity and the induced genomic instability may lead to liver damage and HCC [55, 56].

## Conclusion

This study shown that HBV genotype E and A were the most frequent strain circulating in Congolese patients. HBV genotype E was predominant in all patient groups and genotype A was significant in LC and HCC patients. Different HBV pres1 substitutions found in this study were located in the hepatocyte binding epitope and have possible association with the development of progressive liver diseases in chronic HBV carriers. These data are of paramount importance for decision making in relation to the adoption of treatment strategies and shown the necessity to implement of an effective program to fight HBV infection.

## Abbreviations

ASC, Asymptomatic carriers; CAH, Chronic active hepatitis; LC, Liver cirrhosis; HCC, hepatocellular carcinoma; AST, Aspartate aminotransferase; ALT, Alanine aminotransferase; HBV, Hepatitis B virus; HCC, Hepatocellular carcinoma; HBeAg, Hepatitis B e antigen; HBeAb, Hepatitis B e antibody
